# Improved depressive symptoms in patients with refractory Gilles de la Tourette syndrome after deep brain stimulation of posteroventral globus pallidus interna

**DOI:** 10.1002/brb3.2635

**Published:** 2022-05-27

**Authors:** Aijun Liu, Yongcheng Jiao, Shaohui Zhang, Haibo Kong

**Affiliations:** ^1^ Department of Neurosurgery the Chinese PLA General Hospital Haidian Beijing China

**Keywords:** deep brain stimulation, internal globus pallidus, neuromodulation, Tourette syndrome

## Abstract

**Objective:**

Deep brain stimulation (DBS) has been used on drug‐resistant Gilles de la Tourette syndrome (GTS) for more than two decades until now, but the stimulating targets are still under exploration until now. In this study, the authors reported the efficacy of the bilateral posteroventral globus pallidus interna (GPi) DBS on tic severity and neuropsychiatry symptoms of seven individuals with GTS.

**Method:**

Seven patients with drug‐resistant GTS were enrolled in this study. The severity of these patients was evaluated with Yale Global Tics Severity Scale (YGTSS), Yale Brown Obsessive Compulsive Scale (YBOCS), Hamilton Depression Rating Scale (HAMD), Hamilton Anxiety Rating Scale (HAMA), and Global Assessment of Functioning Scale (GAF). Bilateral posteroventral GPi were selected as the permanent stimulating targets. Follow‐up period was at least 5 years after surgery in the enrolled patients.

**Results:**

After surgery, one patient reported no improvement during the follow‐up period, and a device removal surgery was performed. The other six patients reported minor to significant improvement. The overall YGTSS, YBOCS, HAMA HAMD, and GAF scores of these patients were changed positively after surgery, but only the improvement of the motor tic and HAMD scores had a statistical difference. No surgical complication was reported.

**Conclusions:**

Bilateral posteroventral GPi DBS could relieve the motor tics and depressive symptoms of the enrolled patients significantly, but the vocal tics and other psychiatric symptoms presented a progression without statistical difference during the follow‐up period. The results of this study suggested that bilateral posteroventral GPi are effective targets for the motor tics in GTS patients, especially with prominent depressive symptoms.

## Graphical abstract

Bilateral posteroventral GPi DBS could relieve the motor tics and depressive symptoms of drug‐resistant Gilles de la Tourette syndrome.

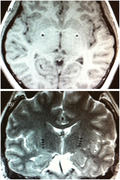

## 1 INTRODUCTION

Gilles de la Tourette syndrome (GTS) is a chronic neuropsychiatric disorder characterized by multiple motor and vocal tics and usually associated with mood and behavioral problems. Most patients with GTS have an onset between 3 and 8 years old, and when they are in their 20s still suffering from the tics regardless of the treatment, up to 90% of these patients will have GTS in their whole life (Pappert et al., 2003). Adult patients with GTS always have many difficulties in the social and family life, and unfortunately, considerable portion of them do not respond well to the pharmacotherapy (Cheung et al., 2007). Some patients even have self‐injurious behavior (SIB), uncontrollable violence, and suicidal behavior (SB) (Cheung et al., 2007; Costanza et al., 2020). Surgical intervention is a reasonable option for these patients aiming at a better life quality, and some surgical procedures have been used to treat the patients with refractory GTS including the ablative surgeries (Babel et al., 2001; Robertson et al., 1990) and deep brain stimulation (DBS) at the selected targets (Ackermans et al., 2010; Bajwa et al., 2007; Burdick et al., 2010; Dehning et al., 2008; Kuhn et al., 2007; Marceglia et al., 2010; Martinez‐Torres et al., 2009; Neuner et al., 2010; Okun et al., 2013; Porta et al., 2009; Servello et al., 2010, 2009; Vandewalle et al., 1999; Welter et al., 2008).

Before the DBS era, we had used the ablative surgeries targeting at the thalamic and subthalamic nuclei to treat the patients with refractory GTS as Babel et al. (2001). Most of these patients actually had a decreased severity of their tics, but a portion of them suffered from some permanent complications postoperatively, such as dystonia, dysphagia, and dysphonia (data are not published). Due to the high risks, we conveyed our eyes from the ablative surgeries to DBS, trying to find a safer modality to treat the refractory GTS. Since Vandewalle et al. (1999) published the first case of the thalamic DBS in GTS in 1999, many neurosurgeons have tried this modality targeting at different nuclei, such as centraomedian nucleus (CM) (Ackermans et al., 2006, 2011; Bajwa et al., 2007; Goethals et al., 2008; Okun et al., 2013); centraomedian nucleus‐parafascicular nucleus (CM‐Pf) (Idris et al., 2010; Maciunas et al., 2007; Marceglia et al., 2010; Porta et al., 2009; Schoenberg et al., 2015; Servello et al., 2010, 2009; Welter et al., 2008); substantia periventricularis (Spv) (Ackermans et al., 2011; Bajwa et al., 2007; Goethals et al., 2008), nucleus ventro‐oralis internus (Voi) (Ackermans et al., 2011; Bajwa et al., 2007; Goethals et al., 2008); anterior and posterior Vo (Voa/Vop) (Idris et al., 2010; Marceglia et al., 2010; Porta et al., 2009); nucleus accumbens (NA) (Burdick et al., 2010; Kuhn et al., 2008, 2007; Neuner et al., 2009, 2010; Servello et al., 2010, 2009; Shields et al., 2008; Zabek et al., 2008); anterior limb of internal capsule (ALIC) (Kuhn et al., 2008, 2007; Neuner et al., 2009, 2010; Servello et al., 2009; Shields et al., 2008); subthalamic nucleus (STN) (Martinez‐Torres et al., 2009); and internal segment of the globus pallidus (GPi) (Ackermans et al., 2006; Cannon et al., 2012; Dehning et al., 2008; Diederich et al., 2005; Dueck et al., 2009; Motlagh et al., 2013; Piedimonte et al., 2013; Sachdev et al., 2014; Servello et al., 2010; Shahed et al., 2007; Smeets et al., 2016; Welter et al., 2008). Among them, CM‐Pf and GPi seemed the promising targets with more successful cases. Considering our clinical experience with DBS electrode placement, we preferred the posteroventral GPi as the targets of DBS to treat the refractory GTS. Since 2009, we have performed bilateral GPi DBS on seven consecutive patients with drug‐resistant GTS. After at least 5‐year follow‐up, the efficacy of these patients was evaluated thoroughly.

Based on our previous ablative surgery experience, we supposed the bilateral GPi DBS could achieve the same effectiveness as the ablative surgery while without any permanent complications.

## 2 MATERIALS AND METHODS

Seven patients with severe uncontrollable vocal and motor tics were admitted to neurosurgery department of our hospital. The GTS diagnosis was established matching the criteria of DSM‐IV‐TR and ICD‐10. All patients had a regular pharmacotherapy lasting at least 8 years, including haloperidol or risperidone. Five of seven patients had also taken sertraline or escitalopram to treat the comorbidity of depression and/or obsessive‐compulsive disorders (OCD). The general characteristics of these patients are shown in Table 1.

**TABLE 1:** General characteristics of seven GTS patients

Patient no.	Sex	Age	Illness duration (year)	Comorbidity	Tics and other symptoms	History of drug therapy	Drug therapy duration (year)
1	M	37	30	–	Vocal, motor	Haloperidol, risperidone	8
2	M	18	12	Depression, OCD	Vocal, motor, SIB	Haloperidol, risperidone with sertraline	12
3	M	18	8	Depression, OCD	Vocal, motor, SIB, incontinence when having severe motor tics	Haloperidol, tiapridal, risperidone with sertraline, escitalopram	8
4	F	24	14	Depression, OCD	Vocal, motor	Haloperidol, risperidone, aripiprazole with sertraline	12
5	M	32	21	–	Vocal, motor	Haloperidol, risperidone, aripiprazole	12
6	M	25	16	OCD	Vocal, motor, SIB	Risperidone, aripiprazole with sertraline	10
7	M	24	18	Depression, OCD	Vocal, motor, SIB	Haloperidol, risperidone, aripiprazole with sertraline	9

Abbreviations: F, female; GTS, Gilles de la Tourette syndrome; M, male; OCD, obsessive‐compulsive disorders; SIB, self‐injurious behavior.

Presurgical examination included electroencephalogram, magnetic resonance imaging (MRI), and neuropsychiatric evaluation. The severity of the tics of these patients was determined with Yale Global Tics Severity Scale (YGTSS) by a same neuropsychiatry practitioner. The general neuropsychiatry status of these patients was also evaluated with Yale Brown Obsessive Compulsive Scale (YBOCS), Hamilton Depression Rating Scale (HAMD), Hamilton Anxiety Rating Scale (HAMA), and Global Assessment of Functioning Scale (GAF). Before surgery, the surgical procedures were clearly illustrated to the patients and their relatives, and the informed consents were obtained from the patients. The surgical protocol was approved by the ethic committee of our hospital.

**FIGURE 1:**
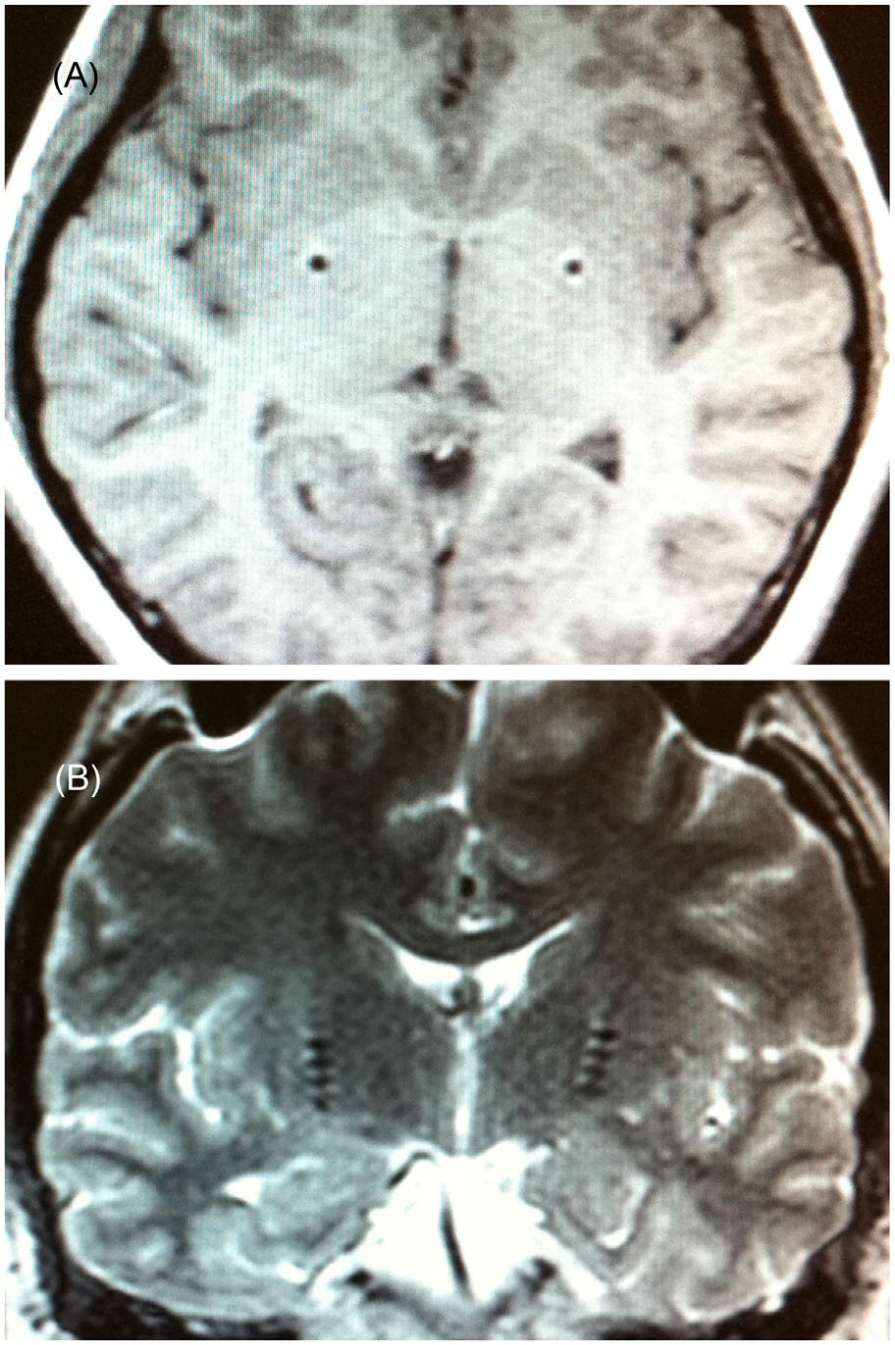
Magnetic resonance imaging (MRI) of axial (a) and coronal (b) slices from one patient showing the trajectory and location of the electrodes implanted bilaterally in the posteroventral globus pallidus interna

We selected the bilateral posteroventral GPi as the targets, and the coordinates were obtained from MRI images with Leksell stereotactic markers. Quadripolar DBS electrodes (Medtronic 3387, Minneapolis, MN, USA) were implanted bilaterally in the posteroventral GPi (20–22 mm lateral, 3 mm below the AC‐PC plane or above the optic tract, and 4 mm anterior to the midcommissural point), and the placement of the electrodes was confirmed by another MRI scan after the electrodes being implanted and fixed (see Figure 1). Then, the electrodes were connected to an infraclavicular pulse generator (Kinetra, Medtronic Inc., Minneapolis, MN, USA). The pulse generator was turned on 2 weeks after the procedures. The stimulation parameters of the DBS device were adjusted intermittently. The postoperative severity of tics was determined with YGTSS, and other neuropsychiatry status was also scored with the same preoperative assessment tools by the same practitioner during the follow‐up period.

## 3 RESULTS

After surgery, all patients were under follow‐up at least 5 years. An every 1‐month clinic visit was recommended to all patients to program the DBS parameters if their symptoms did not improve satisfactorily. The final stimulation parameters are listed in Table 2. One of these patients who had SIB (head hitting and mouth poking) and urinary incontinence occurring with a severe motor tic reported a significant improvement of the tics and a disappearance of SIB and the incontinence. Five of these patients reported minor to moderate general improvement during the follow‐up period. The only female patient in this series complained no improvement and more psychological burden about the DBS device. A removal surgery was performed on request of this patient to take off the DBS device after a 3‐year DBS therapy. All patients did not report any complications during the follow‐up period. The detailed YGTSS assessment of these patients is listed in Table 3. The postoperative motor tic score decreased significantly compared to the preoperative score (*p* < .05, see Table 4), but postoperative vocal tic score did not have a significant decrease compared to the preoperative score (*p* > .05, see Table 4). Being parallel with the change of the motor tic score, the postoperative average total YGTSS score had a significant decrease compared to the preoperative score (*p* < .05, see Table 4). The postoperative average YBOCS score evaluating obsessive‐compulsive status showed a slight decrease compared to average preoperative score (22.29 ± 10.01 vs. 15.29 ± 7.52), but no statistical difference was found between them (*p* > .05, see Table 4). Anxiety severity of these patient was also improved after surgery (average HAMA score 21.14 ± 6.39 preoperatively vs. 16.29 ± 5.59 postoperatively), but still no statistical difference was found between them (*p* > .05, see Table 4). The depression condition of these patients had a significant improvement postoperatively (average HAMD score 46.29 ± 12.39 preoperatively vs. 32.45 ± 9.13 postoperatively), and there was a statistical difference between them (*p* < .05, see Table 4). General condition of these patients was evaluated with GAF, and the postoperative GAF score was increased slightly (52.86 ± 20.79 preoperatively vs. 62.86 ± 15.24 postoperatively, *p* > .05, see Table 4), presenting a mild overall benefit of the DBS therapy in these patients.

**TABLE 2:** Final stimulation parameters of seven GTS patients

	Left electrode contacts					Right electrode contacts					Voltage (V)			
Patients	0	1	2	3	Left case	4	5	6	7	Right case	Left	Right	Pulse (μs)	Frequency (Hz)
1		–			+	–				+	3.6	4	120	130
2					+			–		+	4.2	4	90	130
3		–			+		–			+	5.4	4.4	90	130
4					+		–			+	4	3.8	90	130
5		–			+			–		+	4.2	4.2	90	130
6		–			+		–			+	4.4	4.2	90	130
7					+			–		+	4	4.6	90	130

**TABLE 3:** YGTSS details of seven GTS patients

		Pre‐YGTSS				Post‐YGTSS			
No.	Follow‐up duration (year)	Motor	Phonic	Overall Impairment	Global Severity Score	Motor	Phonic	Overall impairment	Global Severity Score
1	8	18	6	30	54	15	6	30	51
2	7	23	20	50	93	16	16	40	72
3	7	22	20	50	92	17	16	40	73
4	5	23	17	40	80	22	17	40	79
5	8	19	19	40	78	16	18	40	74
6	6	18	17	40	75	16	19	40	75
7	5	22	15	40	77	20	13	40	73

Abbreviations: GTS, Gilles de la Tourette syndrome; YGTSS, Yale Global Tics Severity Scale.

**TABLE 4:** Summary of evaluation scales of seven GTS patients

Test	Preoperation	Postoperation	*p*
**Tic severity (YGTSS)**			
Motor tic	20.71 ± 2.43	16.29 ± 5.32	.006
Vocal tic	17.43 ± 2.53	15.00 ± 4.04	.175
Impairment	41.43 ± 7.53	38.57 ± 3.78	0.5^a^
Total	78.43 ± 14.19	71.00 ± 9.11	.031^a^
OCD (YBOCS)	22.29 ± 10.01	15.29 ± 7.52	.063
Anxiety (HAMA)	21.14 ± 6.39	16.29 ± 5.59	.07
Depression (HAMD)	46.29 ± 12.39	32.45 ±9 .13	.004
General conditions (GAF)	52.86 ± 20.79	62.86 ± 15.24	.056

*Note*: Pre‐ and postoperation scores of each scale are listed as mean and standard deviation. The differences between pre and postoperation scores are compared by paird t‐test. An alpha of.05 is considered statistically significant. All data are analyzed using SigmaPlot 12.5 (Systat Software, Inc.).

^a^Normality test failed, Wilcoxon signed rank test was used.

Abbreviations: GAF, Global Assessment of Functioning Scale; GTS, Gilles de la Tourette syndrome; HAMA, Hamilton Anxiety Rating Scale; HAMD, Hamilton Depression Rating Scale; OCD, obsessive‐compulsive disorders; YBOCS, Yale Brown Obsessive Compulsive Scale; YGTSS, Yale Global Tics Severity Scale.

## 4 DISCUSSION

Drug‐resistant GTS is a challenging disorder for functional neurosurgeons. Fortunately, a few successful treatments of GTS by DBS on different targets have been presented over the last decade; meanwhile, unsuccessful cases were also reported at the same period (Burdick et al., 2010; Motlagh et al., 2013). Generally speaking, the effectiveness of DBS therapy is encouraging in some selected GTS patients, but the use of DBS in GTS is still not as so mature as that in the Parkinson's disease and essential tremor. Only one GTS patient in this study presented a significant general improvement including tics and comorbid mood problems, and the other six patients had only a partial improvement or no improvement. Additionally, no complication occurred in these patients after surgery attributing to the low‐risk nature of DBS procedures. It was worth noting that most of these patients (6/7) had a significant improvement in depressive problem after DBS on bilateral posteroventral GPi, as other authors reported previously in GTS treatment (Cannon et al., 2012; Sachdev et al., 2014). But the DBS therapy did not improve the other mood problems significantly such as anxiety and OCD in these patients. Hoping more improvement after the DBS implantations, we increased the clinic visit frequency of these patients to program the DBS devices. The average frequencies of clinic visit among the seven patients were 10 times per year during the first 2 years after surgery. According to our experience, the improvement of these patients’ symptoms was mainly achieved in the first year after surgery as the other author reported (Sachdev et al., 2014). No matter how frequent to program the device after the first year, the progress of DBS therapy in these GTS patients was minimal.

Patient selection of the DBS therapy for GTS is the first important factor for a satisfactory outcome, but the inclusion criteria are still under discussion at present. Mink et al. (2006) recommended that DBS candidates should be over 25 years old and with YGTSS total tic score more than 35/50. Considering the severity of the young patients included in this series, we extended the inclusion age to more than 18 years old as other authors reported (Maciunas et al., 2007). Martino et al. (2021) also pointed out that the DBS therapy could be considered for the severe younger patients even with age below 18 years. Another study involving 56 adult GTS patients with age over 20 years who had been under follow‐up since the age of 8 to 14 years, demonstrated that up to 90% of those patients still had tics (Pappert et al., 2003). The result of this study provided evidence that early treatment with DBS for a younger patient with severe GTS is reasonable. Moreover, our two younger patients showed better results compared to the other five patients.

The choice of brain targets is another important factor for a successful DBS therapy. CM‐Pf and GPi DBS had the largest population among GTS patients receiving neuromodulation therapy. But both targets had a few unsuccessful cases (Motlagh et al., 2013). And when GPi as a target, different regions of this nuclei such as anteromedial GPi (Cannon et al., 2012; Sachdev et al., 2014; Smeets et al., 2016), posteroventral GPi (Dehning et al., 2014), and even globus pallidus externus (GPe) (Piedimonte et al., 2013) have been used in treatment of GTS. In this study, bilateral posteroventral GPi were selected as the targets and the results demonstrated significant improvement of motor tics and depressive status. Vocal tics and the other psychiatric comorbidity did not improve statistically, only resulting in a mild to moderate improvement. Considering GTS is a complex neuropsychiatric disorder, besides the motor and vocal tics, each GTS patient may suffer from different psychiatric problems with the same YGTSS score. Therefore, the targets for the DBS therapy should be selected based on the patient's dominant symptoms. At present, the limited clinical experience of the DBS therapy for GTS makes it difficult to achieve a satisfactory outcome with a unique target.

Based on the clinical experience from the limited cases, we tended to draw a conclusion that bilateral posteroventral GPi DBS exert a therapeutic effect on the depressive problem in GTS patients. Cannon et al. (2012) reported that bilateral anteromedial GPi DBS also effectively relieved the OCD and depression after 3 months treatment in a clinical study involving 10 patients. Porta et al. (2009) demonstrated that bilateral CM‐Pf DBS could effectively relieve obsessive‐compulsive symptoms, anxiety symptoms, and depressive symptoms involving 18 GTS patients at 2‐year follow‐up. Schoenberg et al. (2015) also reported that bilateral CM‐Pf DBS successfully improved the depression and anxiety symptoms in five male GTS patients in a clinical randomized controlled trial. Okun et al. (2013) performed a clinical trial to study the safety and preliminary efficacy of bilateral centromedian thalamic region DBS on refractory GTS patients. Five patients was enrolled in this study, and the results demonstrated that bilateral centromedian thalamic region DBS did not improve the obsessive and compulsive problems despite the significant improvement in motor and vocal tics. Generally speaking, without the evidence of large randomized controlled clinical study, we could not conclude that GPi or CM‐Pf is an effective target for refractory GTS with psychiatric disorders, but the results of these cases series and our study demonstrated that both GPi and CM‐Pf is a promising target for DBS therapy on the tics and depression problem. Compared to CM‐Pf, neurosurgeons have more clinical experience about the neurophysiological activity and electrodes placement of GPi. Although bilateral posteroventral GPi DBS did not relieve the vocal tic, anxiety, and OCD symptoms in the GTS patients in this study, the motor tics and depression of these patients improved significantly during the long‐term follow‐up period. Our statistical data showed that bilateral posteroventral GPi DBS even produced a stronger effect on the depression symptom than motor tics. In our view, bilateral GPi are the first candidate targets for GTS patients with prominent depression problem if the DBS therapy considered.

The debate about DBS in GTS still exists extensively among neurologists and functional neurosurgeons. Most published papers reported that DBS therapy could relieve the symptoms of the GTS patients, and only few papers presented unsatisfactory cases (Burdick et al., 2010; Motlagh et al., 2013). Given the rarity of the refractory GTS, a publication bias could not be completely avoided. In this study, we reported that after at least 5‐year follow‐up the overall vocal tics, OCD, and anxiety problems of these patients did not improve significantly. More detailed studies should be encouraged to increase the literature size, helping to make the effectiveness of DBS in GTS clear. Besides the effective aspect, a growing concern about suicidal behavior associated with DBS in extrapyramidal diseases (Costanza et al., 2021) should also be noticed when considering DBS in GTS due to the high risk of suicidal behavior in these patients (Fernández de la Cruz et al., 2017; Neuner et al., 2010).

The limitations of this study are obvious. The incidence of GTS in Chinese people is only about 0.01‰, so we just presented our clinical observations based on seven patients and a single target. The severity of these GTS patients was not in the same level, and the prominent symptoms of these patients were also quite different, which made a part of statistical results unreliable. The only one female patient in this study was performed a removal surgery, puzzling us if the gender was a factor to influence the efficacy of DBS in GTS.

## 5 CONCLUSIONS

This study demonstrated that bilateral GPi DBS could significantly relieve the motor tics and depression of GTS patients, but only achieved minor improvement on vocal tics and other psychiatric problems. A drug‐resistant GTS patient with prominent motor tics and depression might be a suitable candidate for bilateral GPi DBS.

## CONFLICT OF INTEREST

The authors declare no conflict of interest, financial or otherwise.

### PEER REVIEW

The peer review history for this article is available at https://publons.com/publon/10.1002/brb3.2635

## Data Availability

The data that support the findings of this study are available from the corresponding author AL, upon reasonable request.
